# External Quality Assessment for Zika Virus Molecular Diagnostic Testing, Brazil

**DOI:** 10.3201/eid2410.180360

**Published:** 2018-10

**Authors:** Sally A. Baylis, Johannes Blümel

**Affiliations:** Paul-Ehrlich-Institut, Langen, Germany

**Keywords:** Zika virus, viruses, World Health Organization, international standard, controls, armored RNA, nucleic acid amplification technique, NAT, NAAT, inactivation, external quality assessment

**To the Editor:** Fischer et al. described an external quality assessment exercise for laboratories in Brazil that perform molecular testing for Zika virus and the development of an armored RNA control material (*1*). Armored RNAs are RNA transcripts synthesized by in vitro transcription; they are encapsulated in a bacteriophage protein coat and are nuclease resistant. In addition to the external quality assessment samples, laboratories were sent vials of the World Health Organization (WHO) International Standard (IS) for Zika virus, which was prepared by using heat-inactivated virus (*2*). Concentrations of WHO ISs are defined in IUs, in this case referring to the viral load, and, because of limitations on number of vials prepared in each batch, they are intended for calibrating secondary standards, including calibrators and run controls in IU facilitating comparison of assays (http://apps.who.int/medicinedocs/documents/s23325en/s23325en.pdf). Secondary standards, such as armored RNAs, traceable in IU (in accordance with ISO 17511:2003), are important complementary reagents to WHO ISs. However, the study by Fischer et al. was missing the calibration of the armored RNA in IU, which is essential for traceability.

Because of packaging limitations of the protein coat, armored RNAs contain only partial genome sequences, compared with live or inactivated virus preparations such as the WHO ISs, which contain full-length genomes. Consequently, armored RNAs are restricted to controlling only certain assays. However, it may be easier to import armored RNAs into countries where disease outbreaks are occurring because they are noninfectious and have not been derived from either viremic plasma or cell culture–derived virus that has undergone heat inactivation. 

Inactivation protocols of viral stock materials must be validated on a case-by-case basis. Certain viruses, such as Zika virus, are heat labile (*3*), whereas viruses such as alphaviruses are much more heat resistant, although there is a wide variation in susceptibility to heat inactivation between different members of the genus (*4*,*5*). The availability of controls, such as armored RNAs, is essential to ensure consistent assay performance on a daily basis and maintain stocks of WHO IS samples for calibration.
